# Heterotopic Ossification following Total Elbow Arthroplasty in a Patient with Parkinson's Disease: Case Report and Literature Review

**DOI:** 10.1155/2020/2068045

**Published:** 2020-03-10

**Authors:** Ajay Shah, Michael Uy, James R. Yan, Moin Khan, Bashar Alolabi

**Affiliations:** ^1^Michael G. DeGroote School of Medicine, McMaster University, Hamilton, Ontario, Canada; ^2^Division of Orthopaedics, Department of Surgery, McMaster University, Hamilton, Ontario, Canada; ^3^St. Joseph's Healthcare Hamilton, Department of Surgery, Hamilton, Ontario, Canada

## Abstract

*Introduction*. Heterotopic ossification (HO) usually develops following surgery or trauma. Risk factors for HO following elbow fractures include delay to surgery (>7 days), floating fractures, and elbow subluxation. Systemic risk factors for HO include male sex; concurrent cranial, neurological, or abdominal injury; high-energy trauma; previous development of HO; and contralateral fracture. To date, no studies have reported on Parkinson's disease (PD) as a risk factor for the development of HO. *Case Presentation*. A 68-year-old female with PD (treated with levodopa-carbidopa) sustained a right closed (OTA type A3) distal humerus fracture and was treated with a total elbow arthroplasty. Postoperatively, development of significant near-ankylosing HO was observed and contributed to significant restriction of elbow motion with activities of daily living. After HO maturation, the osseous growth was excised, and the area irradiated. The patient regained excellent elbow motion with no recurrence of HO. *Discussion*. A literature review revealed six cases of HO development in PD patients following arthroplasty. Patients with PD have higher serum concentrations of interleukins (IL) and tumor necrosis factor- (TNF-) *α*. These factors stimulate BMP-2 production which may promote osteogenesis. Levodopa-carbidopa may also influence HO through stimulation of growth hormone and IGF-1. *Conclusion*. Parkinsonism may promote heterotopic bone growth through the release of osteoinductive factors. HO development may also be mediated by levodopa-carbidopa therapy. Future research should highlight the link between HO and PD and identify if prophylaxis is warranted in PD patients undergoing arthroplasty.

## 1. Introduction

Heterotopic ossification (HO) is defined as the formation of mature lamellar bone in soft tissue sites following surgery or trauma [1]. It is a common complication following fractures, surgical fixation, and arthroplasty. The exact molecular and cellular pathways leading to HO are complex and not yet fully known [2]. It is hypothesized that three components are required for the development of HO: osteoinductive signalling pathways, inducible osteoprogenitor cells, and a heterotopic environment suitable for osteogenesis [3]. If all three components are met, local mesenchymal cells will be recruited to proliferate and differentiate into chondrocytes and osteoblasts, which induces ectopic bone formation [4].

HO formation is initiated by localized inflammation, usually due to injury or iatrogenic trauma [5]. Local soft tissue fibroblasts are induced to transform into pluripotent mesenchymal cells and, ultimately, osteoblasts [6]. Bone is formed via endochondral ossification. This process begins within 16 hours of surgical procedures [7]. Osteoblastic activity has a rate of new bone formation approximately three times that of normal bone in HO, evidenced by increased bone scan activity [8, 9]. Essential to ectopic bone formation is the inductive role of bone morphogenic protein (BMP) signalling [10]. Local overexpression of BMPs and underexpression of antagonists are key for the formation of heterotopic bone [11]. BMPs are stimulated by proinflammatory factors (interleukin-1*β*, prostaglandin E1, and E1) and inhibited by nonsteroidal anti-inflammatory drugs (NSAIDs) [10].

The reported prevalence of HO after elbow fractures ranges from 15 to 37% [12]. Delay to surgery >7 days reportedly results in 10-12 times the odds of developing symptomatic HO [13]. Elbow subluxation, floating fractures, ulnohumeral dislocation, and open fractures are more likely to result in clinically relevant HO [14]. Other risk factors associated with HO after total joint arthroplasty include male sex, concurrent cranial or abdominal injury, intra-articular debris, contralateral fracture, burn injuries, CNS trauma, certain surgical approaches, and development of HO after previous arthroplasty [14]. The optimal classification for upper extremity HO is offered by Hastings and Graham (Table 1) [15, 16]. Pain and a symptomatic reduced range of motion are surgical indications for excision of HO [8]. Surgery should occur after maturation of HO, typically at least 6 months after surgery [6]. Recurrence of HO after excision is common and can be prevented by prophylactic measures including NSAIDs such as indomethacin and perioperative radiation therapy [17].

The purpose of this study is to introduce a case of a patient with Parkinson's disease (PD) who developed severe HO following total elbow arthroplasty in the absence of any other major risk factors. A literature review of cases of HO in PD patients undergoing joint arthroplasty will then be presented, as well as a summary of basic science papers which explore the link between Parkinsonism and ossification.

## 2. Case Report

**Figure 1:**
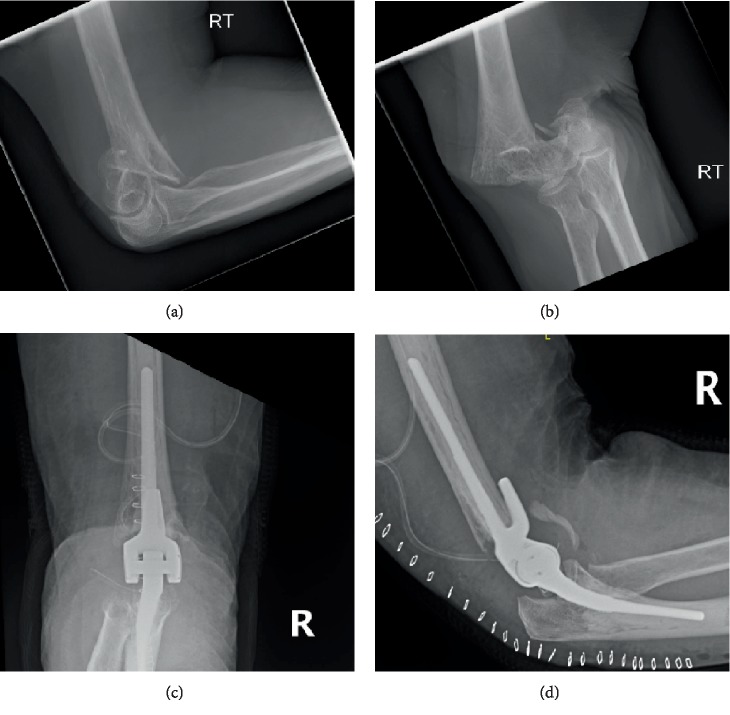
(a, b) Initial X-rays of right distal humerus fracture. (c, d) Immediate postoperative X-rays following total elbow arthroplasty.

**Figure 2:**
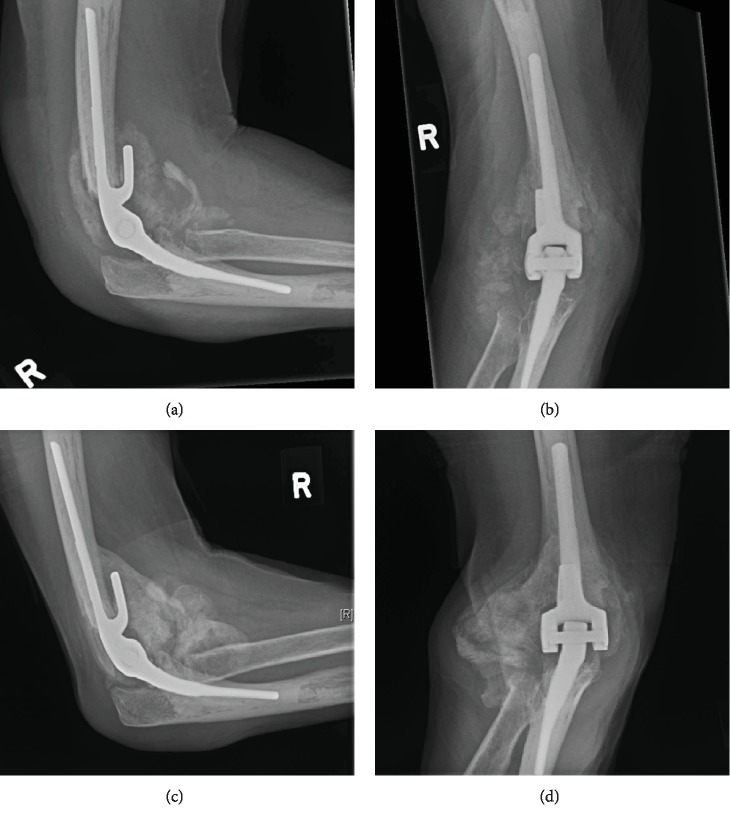
(a, b) Marked heterotopic ossification (HO) seen around the prosthesis at the 6-week follow-up X-rays. (c, d) Maturation of the HO seen at 18 weeks postoperation.

A 68-year-old female with PD sustained a right closed (OTA type A3) distal humerus fracture in her dominant arm. She takes levodopa-carbidopa 150 mg po twice daily for her PD. The patient was treated with a total elbow arthroplasty and an ulnar nerve anterior subcutaneous transposition (Figure 1). Postoperatively, she noticed swelling and gradual increasing stiffness and loss of range of motion (ROM). Her activities of daily living were affected as she was unable to feed herself or adequately perform self hygiene. HO development was noticed radiographically at 6 weeks (Figures 2(a) and 2(b)). ROM at that time was limited to 30° extension to 100° flexion. Initial treatment included physiotherapy as well as dynamic splints.

**Figure 3:**
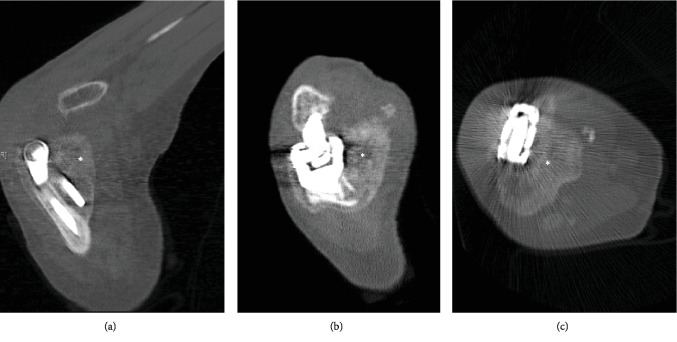
Select computer tomographic views of the elbow in the sagittal (a), coronal (b), and axial (c) planes demonstrating significant HO (marked by the asterisk) engulfing the hardware and native bone.

This patient clinically and radiographically was followed until cortication and maturation of the HO with no change in HO size were noted on consecutive radiographs 6 weeks apart (Figures 2(c) and 2(d)). At this point, her ROM had decreased to 45° extension to 90° flexion despite splinting and physiotherapy, indicating a class IIa lesion by the Hastings classification. CT imaging was then performed to plan for operative excision of the ectopic bone (Figure 3).

**Figure 4:**
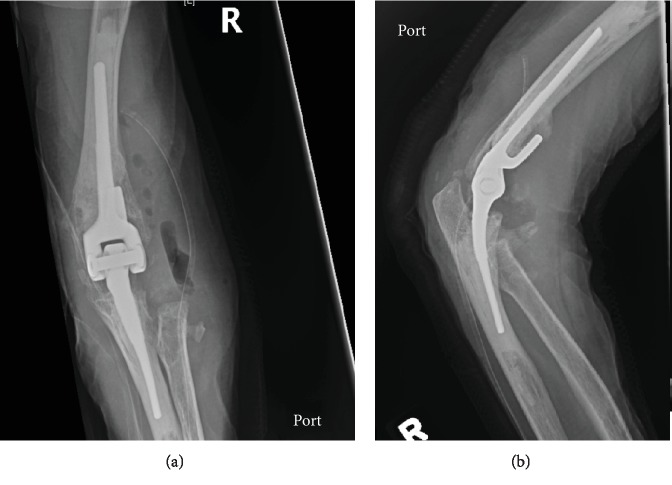
(a) AP and (b) lateral radiographs of R elbow in recovery bay following excision of HO.

An open excision of HO and soft tissue releases (Figure 4) was conducted. A lateral humeral column approach was used with the Kaplan interval (between the extensor digitorum communis and extensor carpi radialis longus) anteriorly and the posterior part of the Kocher interval (between the anconeus and extensor carpi ulnaris). Careful dissection and reflection allowed anterior and posterior access. Extensive bone was removed but the components were secure and not revised. The ulnar nerve was not explored. Intraoperative tranexamic acid was applied locally into the wound to decrease bleeding from the bony surfaces. A drain was inserted during closure to prevent hematoma formation and was discontinued within 48 hours postoperatively.

**Figure 5:**
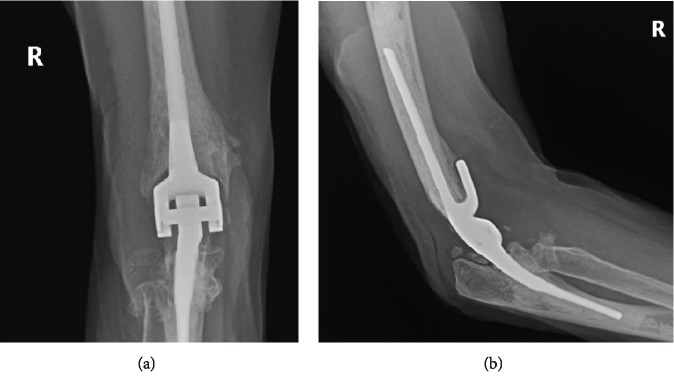
Follow-up AP (a), and lateral (b) X-rays 10 months after HO excision and radiation treatment.

Within 24 hours of the HO excision, the patient received a single fraction of 800 cGy parallel opposed pair beam radiation. Continuous passive motion machine therapy was initiated on the first postoperative day; however, the patient was unable to tolerate the machine due to her PD and it was discontinued. The patient was started on immediate active physiotherapy and dynamic splinting, as well as oral NSAID for 1 month. At 2 weeks follow-up, her ROM was 20° extension to 130° flexion; by 3 months, it was 15° to 140°. At her latest follow-up, 10 months after the excision and radiation treatment, the patient did not have any residual pain and had a ROM from 10° extension to 140° flexion. Her wound was well-healed, and there was no significant recurrence of the HO on follow-up radiography (Figure 5).

## 3. Discussion

The development of HO in this Parkinson's disease patient without any other significant risk factors was notable. The rapid growth and large mass of ectopic bone prompted a literature review to determine if PD could be considered a risk factor for the development of HO. Duffy and Trousdale [18] reported a case of myositis ossificans in a post-TKA PD patient. Two cases of Brooker I and one case of Brooker II HO were reported in post-THA patients with neuromuscular diseases (Table 2) [19, 20]. Pellegrini and Gregoritch [21] reported a case of Brooker III HO progressing to Brooker IV HO following revision THA despite postoperative irradiation. Weber et al. [22] noted that HO was a “rare” complication following THA in PD patients but did not quantify this assertion.

A case report by Ifedi et al. [23] described a 58-year-old man with PD on levodopa-carbidopa who developed HO two weeks after internal fixation of a left closed distal radius fracture. One month later, the patient suffered another low-energy injury, presenting with a right closed distal radius fracture. Again, this fracture was treated with internal fixation and developed HO. The rapid development of HO following low-energy closed fractures provided interesting parallels to this current case. Ifedi et al. [23] hypothesized that the HO was related to either the patient's PD diathesis or levodopa-carbidopa medication.

PD has been described as a pathology associated with higher rates of HO [24]. Pellegrini and Gregoritch [21] state that PD patients are at an increased risk for HO because of systemic diathesis. Namazi [25] suggested a pathway for PD-mediated HO development. Patients with PD were found to have significantly elevated serum concentrations of interleukins (IL) 2, 4, 6, and 10 and tumor necrosis factor- (TNF-) *α* [26]. Peripheral blood mononuclear cells (PBMC) from PD patients had significantly increased secretion of IL-1*β*, IL-6, and TNF-*α* [27]. TNF and IL-1*β* have been shown to stimulate bone morphogenetic protein- (BMP-) 2 production by chondrocytes *in vitro* [28]. BMPs are local factors that induce ectopic syndesmophyte formation, particularly following inflammation in connective tissue [29]. The specific relationship between BMPs and IL-1 has been characterized: IL-1 acts primarily on cell proliferation; BMP primarily shows cell differentiation in the form of HO [30]. Animal studies show that IL-1*β* and BMP increase the total amount of heterotopic bone growth up to sixfold over BMP alone [30]. Interestingly, local BMP-2 levels may still be elevated following HO excision and may predispose to recurrence [31]. The literature did not specify whether systemic cytokine levels (e.g., IL-6 and TNF-*α*) are affected by HO excision.

Another pathway for HO development is through levodopa-mediated processes. Levodopa has been used *in vivo* to promote bone growth after internal fixation of fractures [32]. It was found to stimulate osteogenesis, callus formation, and union via growth hormone (GH) [33]. Levodopa potentiates GH release by stimulating hypothalamic growth hormone-releasing hormone (GHRH) [34]. GH stimulates osteoblast proliferation and activity directly and through IGF-1 [35]. IGF-1 overexpression is found to increase bone growth through osteoblast pathways [36]. Finally, IGF-1 expression causes the activation of the mTOR signalling pathway in mesenchymal stem cells and eventual differentiation into osteoblasts [37].

Prophylaxis against HO in high-risk patients has proven fairly effective [2]. Current prophylactic measures include NSAIDs and radiation therapy. NSAIDs inhibit bone formation by inhibiting the COX-2 enzyme [38]. Ionizing radiation reduces formation of the BMP-2/BMP receptor complex and targets rapidly dividing cells [10]. It can be delivered perioperatively in doses from 0 to 20 Gy and is sometimes given in combination with NSAIDs. It is shown to be not significantly different from NSAIDs in preventing HO and is approximately 200 times more expensive [39]. Failure of these prophylactic measures to prevent debilitating and painful HO formation is an indication for surgical excision. Based on the findings of this review, patients with PD and a previous case of HO should be considered for HO prophylaxis following orthopaedic surgery.

## 4. Conclusion

The case report of a Parkinsonian patient who developed HO without any significant risk factors has been presented. The literature review identified two potential pathways for ectopic bone growth in PD patients. PD systemic diathesis and levodopa-carbidopa therapy have both been shown to increase bone growth. Prophylactic measures should be taken when performing arthroplasty on PD patients taking levodopa. As the steps of HO formation are discovered, more will be learned about the specific relationship between PD and HO.

## Figures and Tables

**Table 1:** Classification of upper extremity heterotopic ossification by Hastings and Graham [15].

Class	Description and management
Class I	Subclinical; observation, physiotherapy, serial radiographs
Class IIa	Limited flexion/extension due to soft tissue or bony impingement; capsulotomy, release, lengthening
Class IIb	Limited pronation/supination due to soft tissue or bony impingement; capsulotomy, release, lengthening
Class IIc	Limited flexion/extension and pronation/supination due to soft tissue or bony impingement; capsulotomy, release, lengthening
Class III	Complete ankylosis with no motion; capsulotomy, release, lengthening

**Table 2:** Brooker classification of HO following TKA.

Class	Description
I	Islands of bone in the soft tissues
II	Bone spurs arising from proximal femur/pelvis with >1 cm of joint space between ends
III	Bone spurs arising from proximal femur/pelvis with <1 cm of joint space between ends
IV	Apparent ankylosis of hip joint
